# Integrative transcriptome and metabolome analysis reveals the mechanism of fulvic acid alleviating drought stress in oat

**DOI:** 10.3389/fpls.2024.1439747

**Published:** 2024-09-19

**Authors:** Shanshan Zhu, Junzhen Mi, Baoping Zhao, Zhaoming Wang, Zhixue Yang, Mengxin Wang, Jinghui Liu

**Affiliations:** ^1^ Coarse Cereals Industry Collaborative Innovation Center, Inner Mongolia Agricultural University, Hohhot, China; ^2^ National agricultural scientific research outstanding talents and their innovation team, Inner Mongolia grassland talents innovation team, Hohhot, China; ^3^ Oat Engineering Research Center of Inner Mongolia Agricultural University, Oat Engineering Laboratory of Inner Mongolia Autonomous Region, Hohhot, China; ^4^ National Center of Pratacultural Technology Innovation (under way)/M-Grass Ecology And Environment (Group) Co., Ltd., Hohhot, China

**Keywords:** oat, drought stress, fulvic acid, phenylpropanoid biosynthesis, glutathione metabolism

## Abstract

Drought stress inhibits oat growth and yield. The application of fulvic acid (FA) can improve the drought resistance of oats, but the corresponding molecular mechanism of FA-mediated drought resistance remains unclear. Here, we studied the effects of FA on the drought tolerance of oat leaves through physiological, transcriptomic, and metabolomics analyses, and identified FA-induced genes and metabolites related to drought tolerance. Physiological analysis showed that under drought stress, FA increased the relative water and chlorophyll contents of oat leaves, enhanced the activity of antioxidant enzymes (SOD, POD, PAL, CAT and 4CL), inhibited the accumulation of malondialdehyde (MDA), hydrogen peroxide (H_2_O_2_) and dehydroascorbic acid (DHA), reduced the degree of oxidative damage in oat leaves, improved the drought resistance of oats, and promoted the growth of oat plants. Transcriptome and metabolite analyses revealed 652 differentially expressed genes (DEGs) and 571 differentially expressed metabolites (DEMs) in FA-treated oat leaves under drought stress. These DEGs and DEMs are involved in a variety of biological processes, such as phenylspropanoid biosynthesis and glutathione metabolism pathways. Additionally, FA may be involved in regulating the role of DEGs and DEMs in phenylpropanoid biosynthesis and glutathione metabolism under drought stress. In conclusion, our results suggest that FA promotes oat growth under drought stress by attenuating membrane lipid peroxidation and regulating the antioxidant system, phenylpropanoid biosynthesis, and glutathione metabolism pathways in oat leaves. This study provides new insights into the complex mechanisms by which FA improves drought tolerance in crops.

## 1 Introduction

Oat (*Avena nuda* L.) is a globally significant food and feed crop. Its grains are abundant in nutrients, including high levels of β-glucan, protein, fat, and soluble dietary fiber. It exhibits remarkable effects on blood sugar regulation and cholesterol reduction, making it an important medicinal crop (Alemayehu et al., 2023). With the increasing focus on nutrition and dietary health, there has been a gradual rise in market demand for high-quality oat raw materials (Singh et al., 2022). Therefore, the development of the oat industry contributes to agricultural growth and farmers’ income and holds great significance in addressing issues related to residents’ imbalanced nutritional intake. China is the primary origin of large-grain naked oats, with a relatively concentrated planting area primarily located in arid and semi-arid regions such as North and Northwest China. These areas are characterized by harsh natural conditions, and as a result of uneven precipitation distribution and the escalating occurrence, frequency, and intensity of droughts attributed to global warming, drought has emerged as the predominant limiting factor for oat production (Tian et al., 2022; Zhang et al., 2022; Salih et al., 2023). Drought and water scarcity triggers a cascade of physiological and biochemical responses in plant morphology (Wang et al., 2022a). For example, drought stress induces the excessive accumulation of reactive oxygen species (ROS), resulting in oxidative damage to cell membranes and chlorophyll degradation, reduced photosynthetic efficiency, and inhibition of crop growth (Muhammad et al., 2021; Peng et al., 2022; Hu et al., 2023), all of which exert a negative impact on plants. Therefore, exploring appropriate methods to enhance the drought tolerance of oat plants is crucial for optimizing their growth and yield (Gao et al., 2018; Bai et al., 2021). The utilization of plant growth regulators is regarded as a proficient and eco-friendly approach to address this issue (Che et al., 2017; Jesmin et al., 2023).

Fulvic acid (FA) is a low-molecular-weight, highly polar active humic substance that contains active organic functional groups and hydrophilic free radicals (Gong et al., 2020). As a growth regulator, it has multiple regulatory effects on plant growth and development (Liang et al., 2024), can stimulate plant growth, enhance leaf photosynthesis, and regulate antioxidant systems (Justi et al., 2019; Chen et al., 2022b). Most importantly, FA is a key factor affecting plant tolerance to abiotic stresses. For example, its strong antioxidant activity helps maintain the homeostasis of ROS to protect plants from the damage caused by environmental stress (Wang et al., 2019). FA can also activate various antioxidant enzymes, such as superoxide dismutase (SOD), catalase (CAT), peroxidase (POD), and polyphenol oxidase (PPO), to reduce oxidative stress in plants (Yu et al., 2024; Hareem et al., 2024). Plant photosynthesis is highly sensitive to drought stress. FA can improve net photosynthesis, increase chlorophyll content and electron transport, and maintain chloroplast ultrastructure, thus alleviating the adverse effects of abiotic stress on photosynthesis (Liu et al., 2022). In addition, studies on its molecular mechanism reveal that FA can resist drought stress through the gene expression involved in the primary metabolism of maize leaves, particularly photosynthesis, carbon fixation, hormone, and osmotic metabolism pathways (Chen et al., 2022a). FA can also enhance the antioxidant defense ability of tea plants under drought stress by regulating ascorbic acid metabolism, glutathione metabolism, and flavonoid biosynthesis (Sun et al., 2020). At present, most research focuses on the drought resistance of FA-induced crops at the physiological level, while the molecular mechanism of fulvic acid-mediated drought resistance in oats remains unclear. Transcriptome analysis is commonly employed to investigate alterations in gene expression levels under diverse biotic and abiotic stresses, thereby enhancing our understanding of the functionality of the gene regulatory networks and signaling pathways in plants (Zhao et al., 2021; Jiang et al., 2023). Similarly, metabolomics has emerged as a novel tool facilitating comprehensive component analysis, proving valuable for identifying changes in metabolites induced by various environmental fluctuations (Zhang et al., 2021). Integrative transcriptomic and metabolomic analyses establish a robust connection between gene regulation and metabolite generation, facilitating the comprehension of plant responses to dynamic environmental changes (Elakhdar et al., 2023; Cui et al., 2023). Scholars have successfully applied these methods to study plant physiology (Li et al., 2023a; Wang et al., 2023a; Dwivedi et al., 2023). Sun et al. (2020) revealed a crosstalk regulation between FA and ascorbate metabolism and flavonoids biosynthesis, contributing to understanding plant drought tolerance. In our previous experiments, we demonstrated the ability of foliar spraying to mitigate the detrimental effects of drought stress on oat seedlings (Zhu et al., 2022). However, the combined transcriptomic and metabolomic analyses of drought tolerance in oats have rarely been reported. In this study, we employed integrative metabolomic and transcriptomic approaches to investigate the effects of FA on overall leaf responses (including morphology, physiology, gene transcription, and metabolite generation) in oat leaves under drought conditions. The objective of the study is to investigate the physiological and molecular mechanisms by which FA regulates oats under drought stress. Our findings enhance the understanding of FA regulation mechanisms in oat plants and provide valuable strategies for improving crop drought tolerance.

## 2 Materials and methods

### 2.1 Plant material and experimental treatments

The drought-sensitive oat variety ‘Bayou 9’ was used in this investigation, characterized by a fertility period of approximately 80 to 90 days. Thirty seeds were sown into plastic pots of 25 centimeters in diameter and 18 centimeters in depth, each containing roughly 3 kilograms of mixed soil (soil, vermiculite, peat soil in a volume ratio of 1:1:1). By the third leaf stage, each pot was thinned to 20 seedlings. The seedlings were grown in a controlled greenhouse at Inner Mongolia Agricultural University (Hohhot, China) at a temperature of 20 ± 2°C, under a photoperiod of 16 hours of light and 8 hours of darkness, with a light intensity of 2000 lux. All pots were at 75% water prior to the jointing stage; water content was maintained by weighing every two days and applying water as needed. Drought stress (water content maintained at 45%) was applied to the seedlings at the jointing stage, and the stress persisted until maturity.

Three treatments were implemented, each with three replicates (pots): (1) spraying distilled water and watering to 75% field capacity (CK); (2) spraying distilled water and exposed to drought stress (DS); and (3) spraying FA and exposed to drought stress (DF). Sprinkle FA once during the plant’s early jointing, heading, and filling stages, at a rate of 15 ml per pot. The FA concentration (600 mg/L) used in this investigation was based on our previous research (Zhu et al., 2022). After 7 days of FA treatment (7 days based on the previous research of the research group) (Li et al., 2019), flag leaf samples were collected, quickly frozen in liquid nitrogen, and stored at -80°C for future measurements.

### 2.2 Growth analysis

The flag leaf, penultimate leaf, and third to last leaf of the oat plant were selected as the study objects. The length and width of the leaves were measured with a ruler. The leaf area was calculated based on the length–width coefficient method:


leaf area=leaf length×leaf width×0.73

where 0.73 is an empirical coefficient. The chlorophyll content of the flag leaves was determined using a chlorophyll meter (SPAD-502, Konica Minolta, Tokyo, Japan). Dry matter quality was estimated by taking the flag leaf, penultimate leaf, and third of 20 oat plants were put into paper bags, and then put into oven at 105°C for 20 min and subsequently dried at 80°C for 48 hours to constant weight. The relative water content (RWC) of leaves was determined by the drying and weighing method (Farooq et al., 2009). Briefly, the fresh weight (W1) of oat flag leaves was weighed and the leaves were then placed on a water surface for 12 h to obtain the saturation weight (WS). Finally, the leaves were dried at 85°C to a constant weight (W2). The RWC of the oat leaves was calculated as follows:


RWC (%)=(W1−W2)/(WS−W2)×100

### 2.3 Measurements of physiological characterization

Malondialdehyde (MDA) content was determined by thiobarbituric acid (TBA) colorimetric method. superoxide dismutase (SOD), peroxidase (POD), catalase (CAT), phenylalanine aminolyase (PAL) activities, hydrogen peroxide (H_2_O_2_), 4-coumaroyl coenzyme A ligase (4CL), and dehydroascorbic acid (DHA) were all measured by enzyme-linked immunosorbent assay kit instruction (Keshun Biotechnology Co., Ltd., Shanghai, China).

### 2.4 RNA extraction, cDNA library construction, and transcriptome sequencing

Total RNA was isolated using the mirVanaTM miRNA isolation kit, following to the manufacturer ‘s instructions. The Tru Seq Stranded mRNA LT Sample Prep Kit was used to create the sequencing library, following to the manufacturer ‘s instructions.

The Agilent 2100 Bioanalyzer (Agilent, Santa Clara, CA, USA) was used to determine the library size and purity of the sample (1 μL). Transcriptome sequencing was carried out by OE Biotechnology Co., Ltd. (Shanghai, China). Trimmomatic software was used to handle raw data, perform quality control, remove the adaptor, and filter to obtain clean reads (Bolger et al., 2014). To get the reference genes, clean reads were aligned to the reference genome using hisat2 (Kim et al., 2015). All sequencing readings were uploaded to the National Center for Biotechnology Information (SRA accession number: PRJNA1111847).

### 2.5 Gene annotation, differential expression, and enrichment analyses

The gene function was annotated based on the following databases: Gene Ontology (GO) (http://www.geneontology.org/) and Kyoto Encyclopedia of Genes and Genomes (KEGG) (http://www.genome.jp/kegg/). Cufflinks software was utilized to calculate the fragments per kilobase of transcript per million mapped reads (FPKM) value for each gene, while htseq-count was employed to obtain the read counts of each gene. Differentially expressed genes (DEGs) were identified using the DESeq R package by estimating size factors and performing a negative binomial test. Significantly differential expressions were determined by applying the thresholds p-value ≤ 0.05 and |log_2_FC| ≥ 1. Hierarchical cluster analysis was conducted on DEGs to explore patterns in gene expression. GO and KEGG pathway enrichment analyses were performed on the DEGs using R software version 3.6.2 with employing the hypergeometric distribution.

### 2.6 Metabolite extraction

The samples (80 mg) were meticulously transferred to a 1.5 mL Eppendorf tube, followed by the addition of two small steel balls. An internal standard solution of L-2-chlorophenylalanine (0.3 mg/mL) dissolved in methanol was added at a volume of 20 μL, and each sample received a mixture of methanol and water (7/3, v/v) at a volume of 1 mL. Subsequently, the samples were placed at −20°C for 2 min. The entire sample was then subjected to ultrasonication in an ice-water bath for 30 min and subsequently cooled in a refrigerator at −20°C for 20 min. After centrifugation at 4°C (13,000 rpm) for 10 min, supernatant aliquots of 150 μL were collected from each tube using a crystal syringe and filtered through a microfilter with a pore size of 0.22 μm before being transferred to LC vials. These vials were stored at −80°C until liquid chromatography-mass spectrometry (LC-MS) analysis was performed. A quality control (QC) sample was prepared by pooling all individual samples into one mixed sample.

LC-MS analysis was conducted using a UHPLC system (Vanquish, Thermo Fisher Scientific, Waltham, MA, USA) coupled to a Q Exactive HFX mass spectrometer (Orbitrap MS, Thermo Fisher Scientific) with a UPLC BEH Amide column (2.1 mm × 100 mm, 1.7 μm). The mobile phase consisted of 25 mmol/L ammonium acetate and 25 mmol/L ammonia hydroxide in water (pH = 9.75) (A) and acetonitrile (B). Gradient elution was employed for the analysis as follows: 0–0.5 min, 95% B; 0.5–7.0 min, 95%–65% B; 7.0–8.0 min, 65%–40% B; 8.0–9.0 min, 40% B; 9.0–9.1 min, 40%–95% B; 9.1–12.0 min, 95% B. The column temperature was maintained at a constant value of 30°C throughout the analysis period, while the auto-sampler temperature was set at −4°C and the injection volume used was fixed at exactly 2 μL. MS spectra were obtained in information-dependent acquisition mode using Xcalibur (Thermo Fisher Scientific) and a QE HFX mass spectrometer equipped with an electrospray ionization source. Measurements were made under the following conditions: sheath gas flow rate of 50 Arb, auxiliary gas flow rate of 10 Arb, capillary temperature of 320°C, full MS resolution set at 6000 resolving power, MS/MS resolution set at 7,500 resolving power with collision energy values in the range 10–60 under normalized collision energy mode depending on specific requirements during the data acquisition process, and positive or negative spray voltage (3.5 kV or −3.2 kV, respectively).

### 2.7 Data preprocessing and statistical analysis

The raw data were preprocessed and normalized using the metabolomics processing software Progenesis QI v3.0 (Nonlinear Dynamics, Newcastle, UK). The Human Metabolome Database (HMDB), Lipidmaps (v2.3), METLIN, and PMDB database were utilized for the qualitative analysis. The selected data were scored according to the qualitative results of the compound with a standard score of 36 points, and data with a score below 36 points were deleted. The resulting positive and negative ion data were combined into a data matrix table. First, unsupervised principal component analysis (PCA) was used for multivariate statistical analysis to observe the overall distribution among the oat samples and the stability of the whole analysis process. Supervised partial least squares-discriminant analysis (PLS-DA) and orthogonal analysis. Partial least squares analysis (OPLS-DA) was used to distinguish overall differences in metabolic profiles between groups and to find inter-group differences. Differential metabolites. Student’s t-test and fold change analysis were employed. To compare metabolites between the two groups. The method of combining multidimensional analysis and one-dimensional analysis was adopted. The following thresholds were used for the analysis: VIP value of the first principal component of the OPLS-DA model > 1; FC > 1; and t-test p-value < 0.05. Quasi-screening was adopted for differential metabolites between groups. Functional annotation and pathway analyses of differential metabolites were performed using KEGG database enrichment analysis.

### 2.8 Real-time quantitative reverse transcription PCR analysis

To verify the reliability of the DEGs from the sequencing results, eight DEGs were selected for internal q-PCR. The specific primers are reported in **Supplementary Table 1**, with the oat *actin* gene employed as the internal reference gene. The RNA was reverse transcribed into cDNA using TransScript All-in-One First-Strand cDNA Synthesis SuperMIX for q-PCR (All-in-One Gold). q-PCR was performed on an ABI9700 system using the dye method. The reaction system, described in **Supplementary Table 2**, was executed under the following conditions: 94°C for 30 s; 40 cycles of 94°C for 5 s; and 60°C for 30 s. The temperature was slowly increased from 60°C to 97°C, and the fluorescence signal was collected five times per °C. Each sample was replicated three times, with sterile water used as the control. The relative expression of the genes was calculated with the 2^-ΔΔCt^ algorithm.

## 3 Results

### 3.1 Morphological and physiological responses of the oat plant induced by FA under drought stress

**Figure 1:**
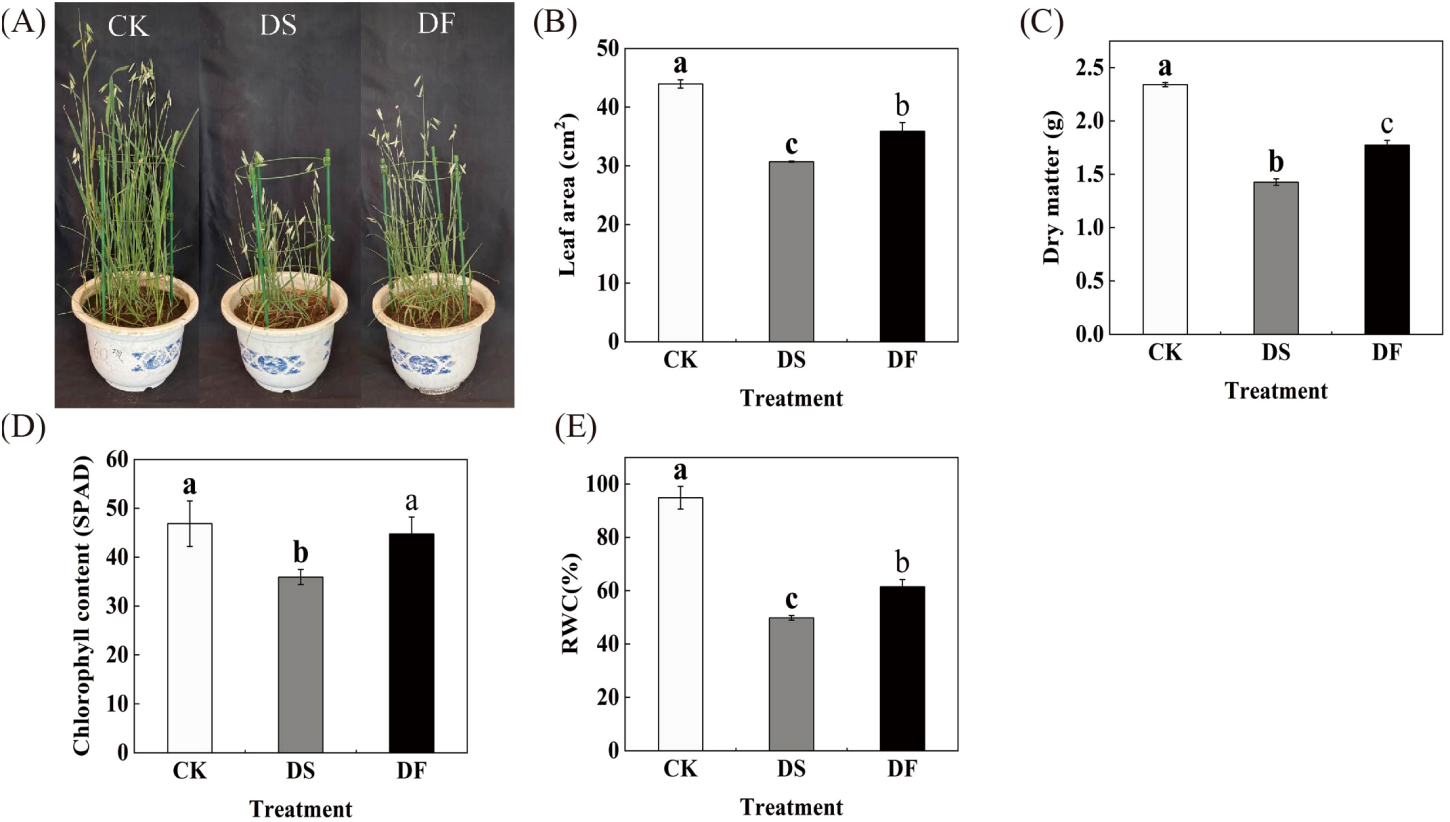
Effects of fulvic acid on the morphology and physiology of oat (*Avena sativa* L.) under drought stress. CK was the control, DS was exposed to drought stress, and DF was exposed to drought stress and was treated with fulvic acid. **(A)** growth condition. **(B)** chlorophyll content. **(C)** leaf area. **(D)** dry weight. **(E)** relative water content. The columns represent the means and the error bars represent ± SD of three replicates. Columns in the same panel and capped with the same lower case letter are not significantly different *P* > 0.05 according to Duncan’s multiple range tests.

We investigated the morphological and physiological changes in FA-treated leaves under drought conditions to confirm the positive effects of FA in alleviating drought stress in oat leaves. Under drought stress, the oat plants exhibited a significant reduction in height, accompanied by a decrease in leaf count. The lower leaves displayed green and yellow discoloration and curling, while the upper leaves showed scorching at the edges. Additionally, there was a notable decline in spikelet numbers. The number of dead leaves, the length of dead leaves, and the degree of yellowing all exhibited a significant reduction in FA-treated leaves under drought stress in comparison to the control group. There was also an observed increase in the number of spikelets (**Figure 1A**). The leaf area and dry matter quality of the oat leaves were significantly reduced under drought stress, while the application of FA mitigated the detrimental effects of drought stress on these parameters (**Figures 1B, C**). Under drought stress, the chlorophyll content and RWC declined by 23.29% and 47.48%, respectively, compared to the control, while the application of FA resulted in a significant recovery of chlorophyll content and RWC by 24.5% and 23.33%, respectively (**Figures 1D, E**). These results indicate that FA alleviates the negative effects of drought on oat leaves and promotes plant growth.

**Figure 2:**
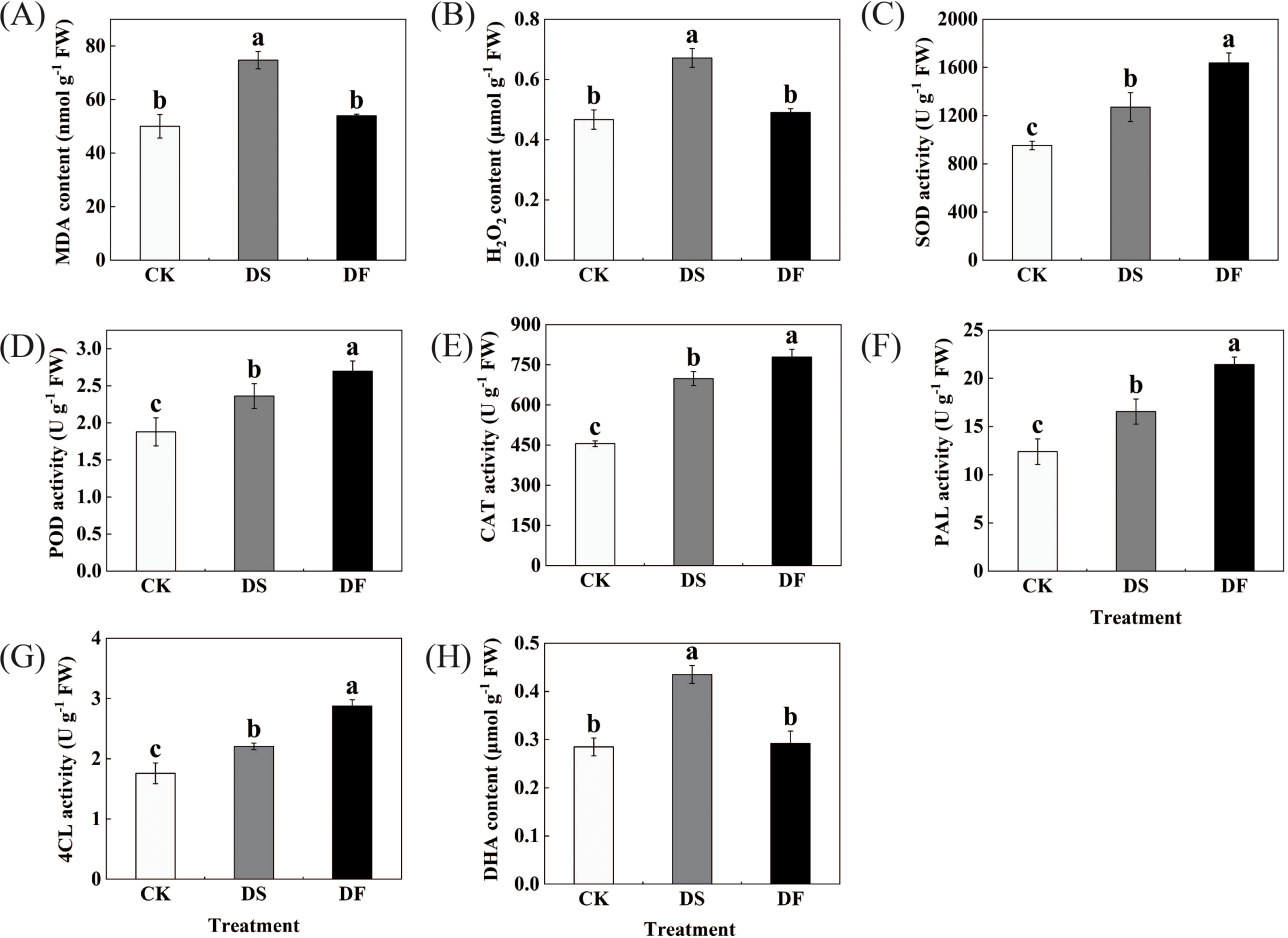
Effects of Fulvic acid on **(A)** MDA content, **(B)** H_2_O_2_ content, **(C)** SOD activity, **(D)** POD activity, **(E)** CAT activity, **(F)** PAL activity in oat,**(G)** 4CL activity, and **(H)** DHA content (*Avena sativa* L.) leaf under drought stress. CK was the control, DS was exposed to drought stress, and DF was exposed to drought stress and was treated with fulvic acid. The bars represent the means ± SD of three replicates. Different letters indicate significant differences at *P* < 0.05 according to Duncan’s multiple range tests.

MDA and H_2_O_2_ levels were selected as markers of oxidative damage and ROS accumulation. Drought stress resulted in a significant enhancement in the levels of H_2_O_2_ and MDA in the oat leaves, while the application of FA effectively mitigated the accumulation of H_2_O_2_ and MDA (**Figures 2A, B**). We estimated the levels of antioxidative enzymes in the oat leaves under different treatments. The changes in these enzymatic antioxidants exhibited a similar variation trend (**Figures 2C-G**). Under water-deficit conditions, the activities of the antioxidative enzymes (SOD, POD, PAL, CAT, and 4CL) significantly increased in oat leaves, and FA application was able to further improve the levels of SOD, POD, PAL, CAT, and 4CL in the oat leaves. Ascorbic acid is one of the most abundant water-soluble antioxidants and can be used as a ROS scavenger directly against oxidative stress. Drought stress led to an increase in dehydroascorbic acid content in oat leaves, while fulvic acid application decreased its content (**Figures 2H**). These results suggest that the application of FA can enhance antioxidant defense activity and decrease excessive ROS.

### 3.2 Leaf transcriptome profiles in response to different treatments

#### 3.2.1 Transcriptome sequencing analysis

RNA-seq analysis was performed on three biological replicates of leaf samples under CK, DS, and DF treatments. Nine cDNA libraries were prepared and then paired-end sequenced. The raw reads ranged from 44.26 to 49.06 million. After removing low-quality reads, clean reads ranged between 44.16 and 48.96 million. In our libraries, the percentage of clean reads was greater than 99%, and the Q30 values were greater than 90.77%. The mean GC content was determined as 55.14% (**Supplementary Table 3**). The transcriptome sequencing results exhibit high quality and are suitable for the subsequent analysis.

**Figure 3:**
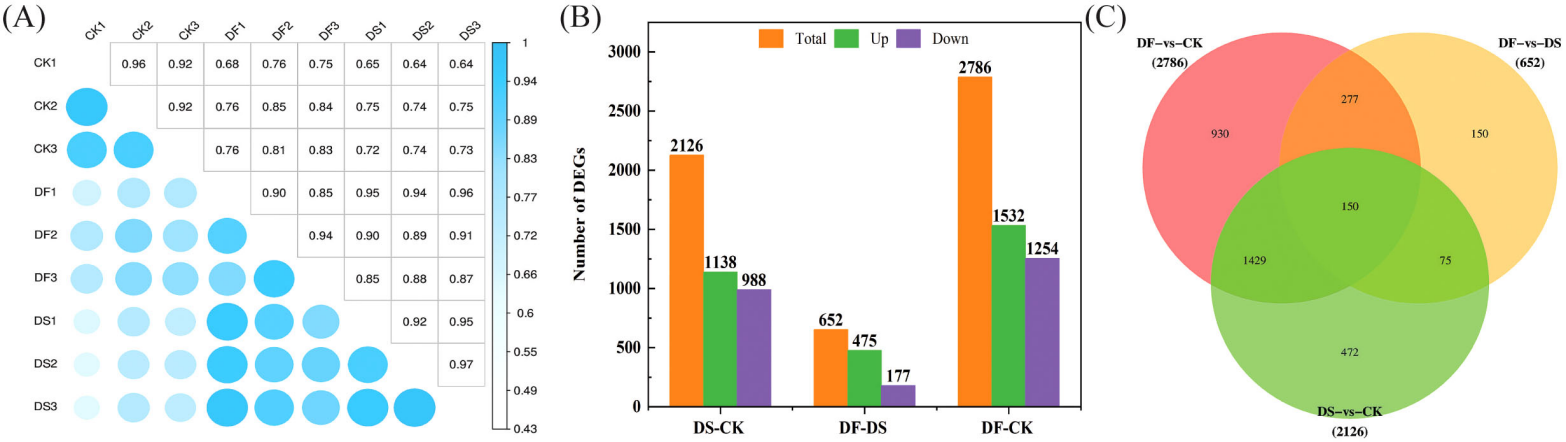
The correlation among samples and the contrast between groups of differentially expressed genes. **(A)** Heat map of correlation coefficient between samples. **(B)** The total number of differentially expressed genes (DEGs) and upregulated and downregulated DEGs under different treatments. **(C)** Venn diagram of DEGs. CK, Normal moisture treatment; DS, drought treatment; DF, Fulvic acid + drought treatment.

Pearson’s correlation coefficient analysis of the three replicates indicated that the sequencing data were of high quality and met the requirements for further analyses (**Figure 3A**). Based on the thresholds of adjusted |log2Fold Change| > 1 and false discovery rate (FDR) of <0.05, a total of 2126, 2786, and 652 DEGs were screened in DS-vs-CK (1138 upregulated and 988 downregulated), DF-vs-CK (1532 upregulated and 1254 downregulated), and DF-vs-DS groups (475 upregulated and 177 downregulated)groups, respectively (**Figure 3B**). There were more upregulated genes than downregulated genes in the seedlings following the DS and DF treatments, and more genes were differentially regulated by drought alone than by the DF treatment. Among the DEGs, 150 transcripts were commonly induced by the CK, DS, and DF treatments. Moreover, we identified 225 common DEGs between the DF-vs-DS and DS-vs-CK groups. These genes are likely to be involved in the alleviation of drought stress in oats through the action of FA (**Figure 3C**). The gene expression trend verified by q-PCR was similar to that determined by RNA-seq (**Supplementary Figure 1**), indicating that the data obtained by transcriptome analysis were highly reliable.

#### 3.2.2 GO and KEGG enrichments of DEGs

**Figure 4:**
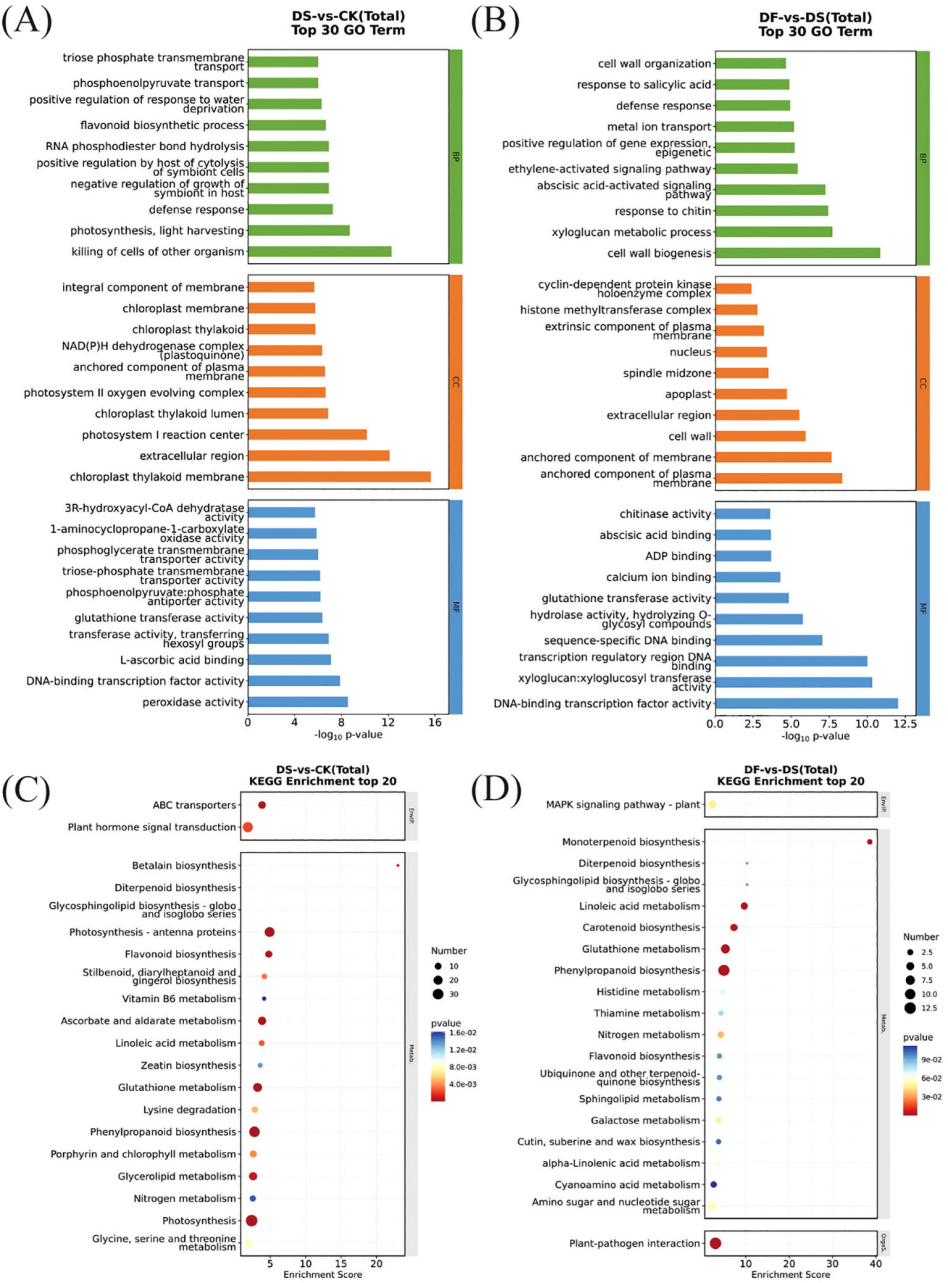
Top 20 enriched GO terms and KEGG pathways from DEGs. **(A)** GO terms of DEGs in DS-vs-CK. **(B)** GO terms of DEGs in DF-vs-DS. **(C)** KEGG pathway analysis of DEGs in DS-vs-CK. **(D)** KEGG pathway analysis of DEGs in DF-vs-DS.

To further understand the functions and the related biological processes of the DEGs, GO enrichment analyses were conducted. The results are presented in **Figures 4A, B**. The DEGs were classified into biological processes, cellular components, and molecular functions. In the biological process of DS-vs-CK, the differential genes were mainly enriched in the defense response and flavonoid biosynthetic process. In the cellular component, the most enriched DEGs were the integral component of the membrane and the chloroplast thylakoid membrane. The molecular function mainly included DNA-binding transcription factor activity and peroxidase activity. (**Figure 4A**). These terms have been reported vital for drought stress tolerance in plants. The most differentially enriched genes of DF-vs-DS in biological processes were the defense response, cell wall organization, and the abscisic acid-activated signaling pathway. The DEGs enriched in cellular components were the nucleus and extracellular region. DNA-binding transcription factor activity and sequence-specific DNA binding were the main types of genes with molecular function enrichment (**Figure 4B**). Thus, genes encoding functionally diverse proteins contribute to the FA-mediated response of oat plants to drought stress, and DF treatment uniquely and differentially regulates genes for multiple functions.

To identify the main pathways by which FA contributes to drought tolerance, the DEGs were subjected to KEGG pathway enrichment analysis. **Figures 4C, D** depicts the scatter plot of enrichment analysis combined with the number of DEGs-identified significant pathways. In total, 785 (DS-vs-CK) and 150 (DF-vs-DS) DEGs were mapped to 108 and 67 KEGG pathways, respectively (**Supplementary Table 4**). The photosynthesis-antenna proteins, phenylpropanoid biosynthesis, photosynthesis, ascorbate and aldarate metabolism, and glutathione metabolism pathways were enriched in the DS-vs-CK group (**Figure 4C**). There were more upregulated genes in the pathway of photosynthesis-antenna protein and photosynthesis than downregulated genes, while the genes in the pathway of phenylpropanoid biosynthesis, ascorbate and aldarate metabolism, and glutathione metabolism showed an opposite trend. This indicates that the photosynthesis of oat leaves in the drought treatment group was weakened, and some secondary metabolites and their biosynthetic pathways were activated to enhance the drought tolerance of oat (**Supplementary Table 5**). Phenylpropanoid biosynthesis and glutathione metabolism pathways were significantly enriched in the DF-vs-DS group (**Figure 4D**), and the number of upregulated genes was greater than that of downregulated genes (**Supplementary Table 5**). This suggests that FA improved the antioxidant defense system and drought resistance of plants, mainly by promoting the expression of phenylpropanoid biosynthesis and glutathione metabolism.

Photosynthesis significantly affected the expression of genes encoding key enzymes in the photosynthesis pathway, including photosystem II, photosystem I, photosynthetic electron transport, and F-type ATPase (**Supplementary Figure 2**). Compared with CK, the genes related to photosynthesis were downregulated under drought stress, with the exception of *PetH*(ferredoxin–NADP+reductases). Similarly, 25 expressed genes related to the light-harvesting chlorophyll (*LHC*) protein complex in photosynthetic antenna proteins were downregulated. However, these same genes—encoding *PsbS* and *Lhcb1*—were upregulated by FA under drought stress.

### 3.3 Leaf metabolome profiles in response to different treatments

**Figure 5:**
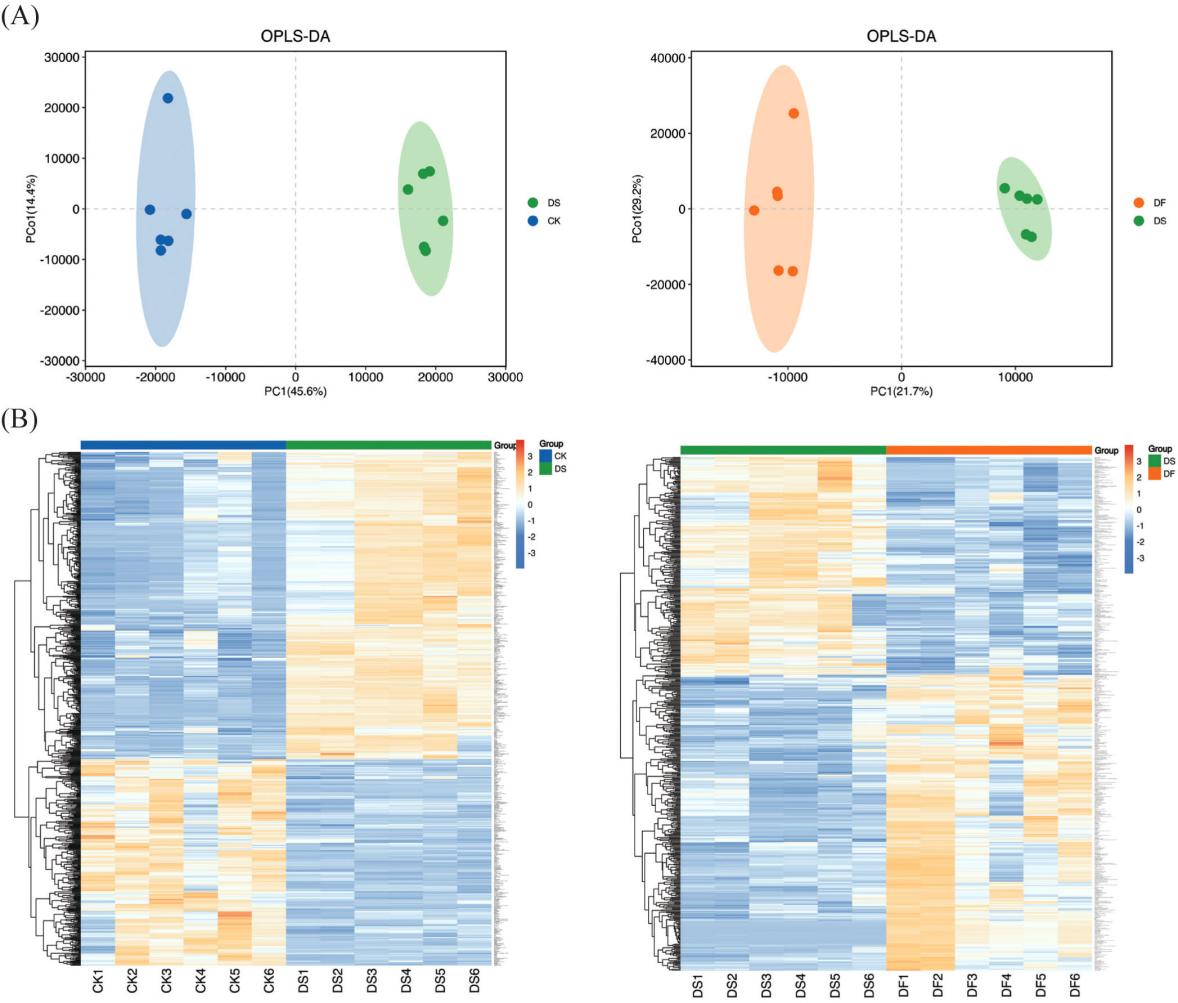
Overview of widely targeted metabolome analysis of leaves responsive to CK, DS, and DF treatments. **(A)** OPLS-DA score map. **(B)** Heatmap visualization of the metabolites. The transition from blue to red indicates a gradient of expression abundance for metabolites, ranging from low to high; the redder the color, the higher the expression abundance of differential metabolites.

The dataset employed in this study is complex as the difference between groups is relatively small while the difference within groups is relatively large. Thus, to obtain more reliable differential metabolites between the control groups, OPLS-DA was used to analyze the data collected from the oat leaf samples. In the OPLS-DA score diagram, the separation of two different treatment samples was obvious, and the intra-group clustering was also evident, indicating that there were different metabolites between groups (**Figure 5A**). The heatmap visualized distinct hierarchical clustering of metabolites obtained in all leaf samples, suggesting that these metabolites have different expression patterns in response to the CK, DS, and DF treatments (**Figure 5B**). These findings confirmed that the reproducibility among the biological samples was sufficient for further analyses.

**Figure 6:**
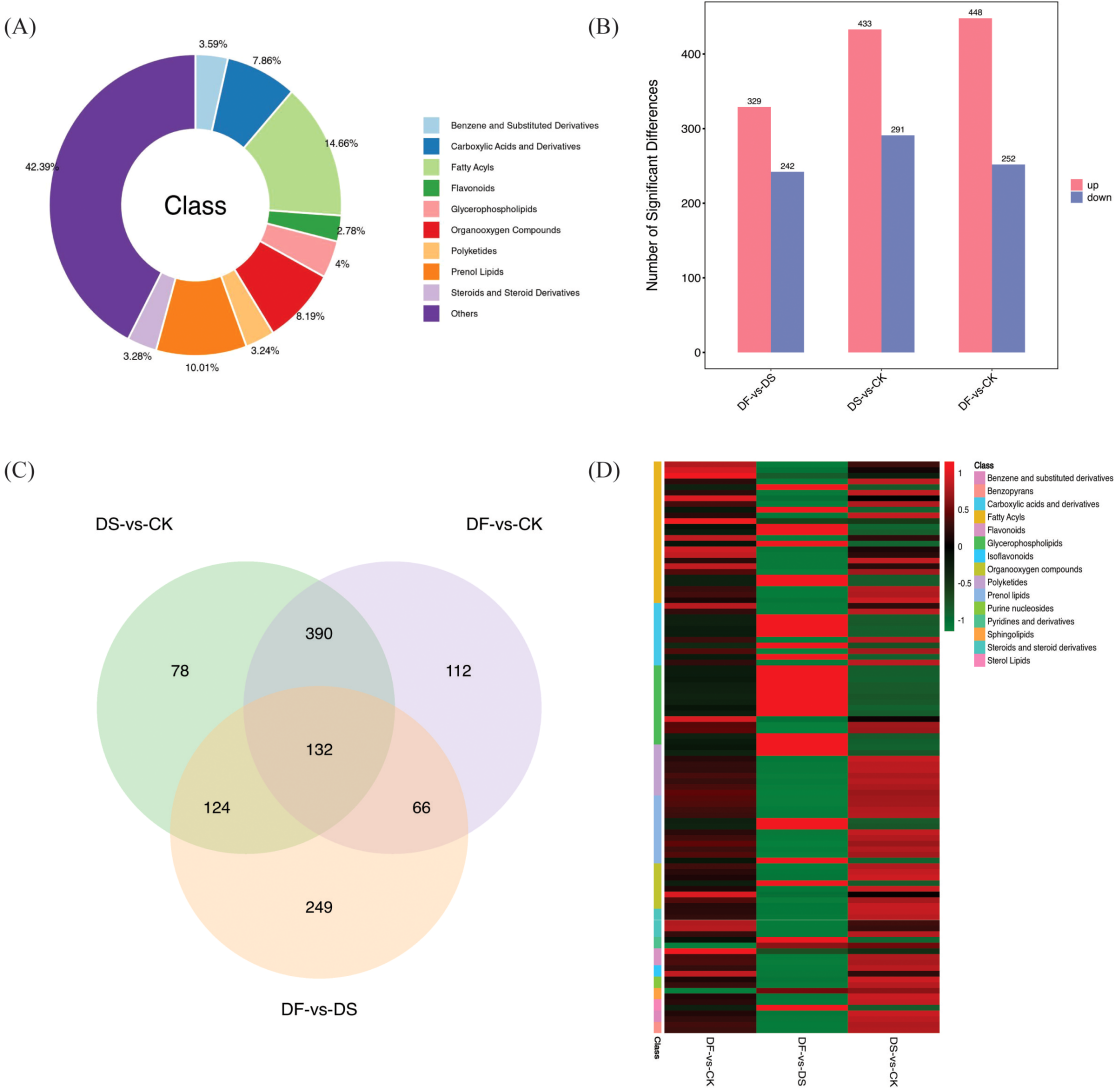
**(A)** Category of the DAMs. **(B)** Number of differential metabolites between different control groups **(C)** Venn diagram illustrating the number of DAMs in DS-vs-CK, DF-vs-DS, and DF-vs-CK groups. **(D)** Heat map analysis of common DEMs in DS-vs-CK, DF-vs-DS, and DF-vs-CK groups.

The significant differences in the relative metabolite content were screened using VIP >1 and p-value <0.05 (t-test results). In total, 8054 DEMs were detected in all leaf samples and were mainly classified into benzene and substituted derivatives (289), carboxylic acids and derivatives (633), fatty acyls (181), flavonoids (224), glycerophospholipids (322), organooxygen compounds (660), polyketides (261), prenol lipids (806), steroids and steroid derivatives (264), and others (3414) (**Figure 6A**; **Supplementary Table 6**). There were 724, 571, and 700 DEGs screened in DS-vs-CK (433 upregulated and 291 downregulated), DF-vs-DS (329 upregulated and 242 downregulated), and DF-vs-CK groups (448 upregulated and 252 downregulated), respectively (**Figure 6B**). Therefore, the differences in metabolite expression induced by FA in oats changed significantly under drought stress. Overlapping analysis showed that 132 metabolites commonly responded to DS-vs-CK, DF-vs-DS, and DF-vs-CK (**Figure 6C**). Interestingly, in the 132 DEMs, fatty acyls, flavonoids, isoflavonoids, and glycerophospholipids were highly accumulated, indicating that FA could improve the drought resistance of oats by regulating the accumulation of lipids and inducing the change of antioxidants (**Figure 6D**; **Supplementary Table 7**).

### 3.4 Integrated analysis of the RNA-seq and metabolome data of oat leaves

**Figure 7:**
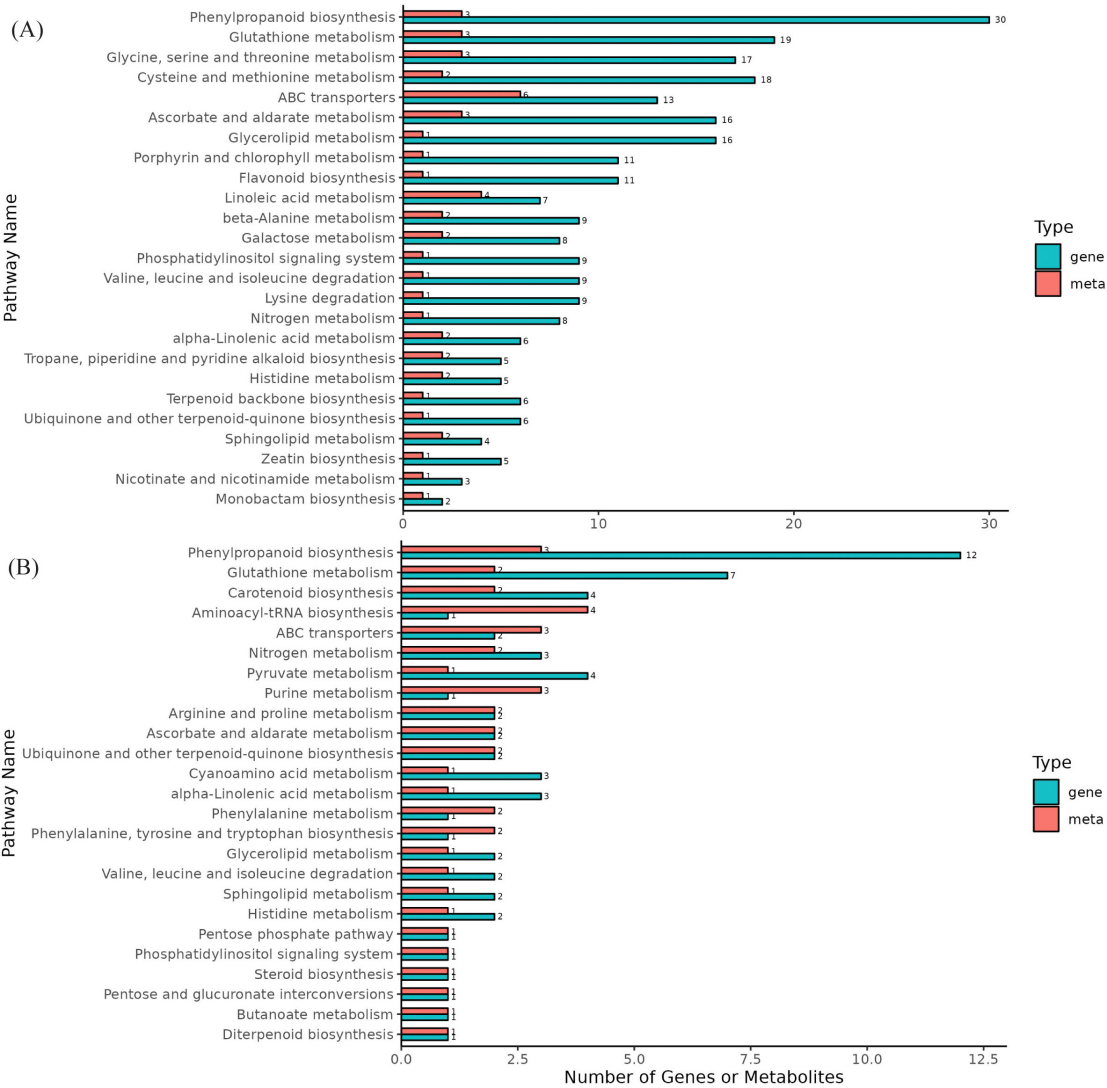
Differential enrichment pathways for metabolites and genes. **(A)**, DS-vs-CK, **(B)**, DF-vs-DS, The horizontal axis represents the number of differentially enriched metabolites and genes in the pathway, and the vertical axis represents the KEGG pathway name. The meta represents the metabolome, and the gene represents the transcriptome.

The DEGs and DAMs from the same group were mapped onto the KEGG pathway database to elucidate the associations between genes and metabolites. A total of 50 and 29 pathways were enriched in DS-vs-CK and DF-vs-DS, respectively, in which genes related to phenylpropanoid biosynthesis and glutathione metabolism pathways were significantly enriched in both control groups, and two metabolic pathways were also enriched in the metabolome (**Figure 7**; **Supplementary Table 8**). Moreover, these two pathways are important for the antioxidant system. Therefore, we focused on the phenylpropanoid biosynthesis and glutathione metabolism pathways.

**Figure 8:**
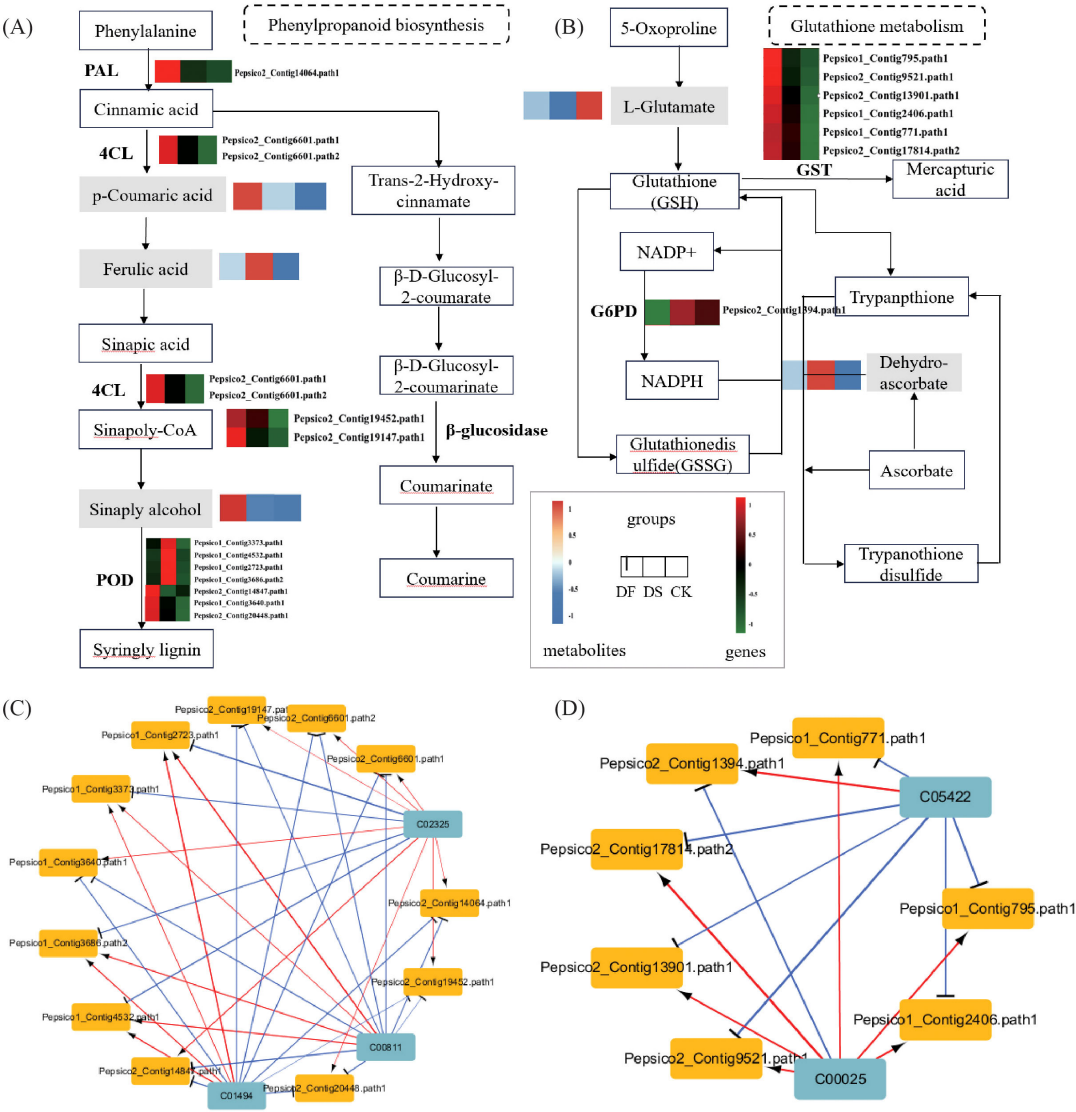
The expression of important pathway genes and metabolites in oat leaves under CK, DS and FA treatments. **(A)** Expression profiles of the genes and metabolites involved in phenylpropanoid biosynthesis. **(B)** Expression profiles of the genes and metabolites involved in glutathione metabolism. The rectangular patterns represent the genes or metabolites, and the heatmap at the corresponding place depicts the differential expression of each identified gene or metabolite, which ranges from green or blue (low) to red (high). **(C)** The related networks of DEGs and DAMs in Phenylpropanoid biosynthesis. **(D)** The related networks of DEGs and DAMs participating in glutathione metabolism and dam. In the related network diagram, blue squares represent metabolites, yellow squares represent genes, red lines indicate positive correlation, blue lines indicate negative correlation, and the thickness of the lines represents the strength of the correlation.

In the phenylpropanoid biosynthesis pathway, two genes encoding phenylalanine ammonia-lyase (*PAL*) and 4-coumarate-CoA ligase (*4CL*) were upregulated, four genes encoding *β-glucosidase* were upregulated and one gene was downregulated, nine genes encoding peroxidase (*POD*) were upregulated and seven genes were downregulated, and the metabolites p-coumaric acid and ferulic acid were upregulated under drought stress. These results indicate that the phenylpropanoid biosynthesis pathway experienced significant changes under drought stress, and the aforementioned genes and metabolites played an important role in protecting oats from drought stress. FA also significantly affected the phenylpropanoid biosynthesis pathway. Most genes were still upregulated under FA, such as *PAL*, *4CL*, *β-glucosidase*, and *POD*. The expression patterns of metabolites p-coumaric acid and ferulic acid were opposite to those under the drought treatment, both of which were downregulated while sinapitol was upregulated. These results indicated that FA could improve the drought resistance of oats by promoting the expression of genes related to the phenylpropanoid biosynthesis pathway and regulating metabolites (**Figure 8A**). In the glutathione metabolism pathway, thirteen genes encoding *GST* were significantly upregulated, the metabolite dehydroascorbic acid was upregulated, and L-glutamate was downregulated under drought stress. FA application increased the expression of six genes encoding *GST* and the level of L-glutamate and decreased *G6PD* and dehydroascorbic acid. These results indicate that FA treatment induced relatively higher expression of glutathione metabolism-related genes and L-glutamate biosynthesis than drought (**Figure 8B**).

Network correlation analysis was conducted on the DEGs and DAMs in these two pathways. In phenylpropanoid biosynthesis, p-Coumaric acid (C00811), and ferulic acid (C01494) exhibited a negative correlation with the genes encoding *4CL*, *PAL*, and *β-glucosidase*, a positive correlation with four genes encoding *POD*, and a negative correlation with three genes. Sinapyl alcohol (C02325) showed an opposite expression pattern with p-Coumaric acid (C00811) and ferulic acid (C01494) (**Figure 8C**). In glutathione metabolism, L-glutamate (C00025) exhibited a positive correlation with genes encoding *GST* and a negative correlation with genes encoding *G6PD*. The expression pattern of dehydroascorbic acid (C05422) was opposite to that of L-Glutamate (C00025) (**Figure 8D**). These findings further confirmed that fulvic acid induced DEGs and DAMs were mainly concentrated in phenylpropanoid biosynthesis and glutathione metabolism pathways, and the differential genes were highly correlated with corresponding metabolites, indicating their importance in FA mediated drought stress response in plant.

## 4 Discussion

Under adverse conditions, FA can promote crop growth and increase yield (Chen et al., 2022b; Jesmin et al., 2023). When plants are subjected to drought stress, the water content in the body decreases and growth is delayed, resulting in a decrease in yield. In contrast, spraying FA can significantly improve plant growth (Shokhmgar et al., 2023). In the present study, under drought stress, spraying with FA was observed to reduce the number of dead oat leaves, deepen the green color of the leaves, and increase the number of spikelets (**Figures 1A-C**). The low molecular weight of FA enables it to penetrate through the pores of the membrane and promote nutrient entry into the cells by forming complexes with cations to increase the leaf area of oat leaves and promote the accumulation of dry matter (Bocanegra et al., 2006; Lalas et al., 2018). As the most important and effective pigment for photosynthesis, chlorophyll can reflect the plant’s ability to assimilate substances to a certain extent, and its content is an important factor in determining the plant’s photosynthetic capacity and substance production (Walker et al., 2018). Leaf RWC reflects the degree of soil aridity as well as the plant’s ability to retain water, and is most relevant to crop drought tolerance (Utsumi et al., 2019). (**Figures 1D, E**). Consistent with this, the expression levels of genes involved in chlorophyll synthesis were changed by FA (**Supplementary Figure 3**). FA decreased the expression levels of genes involved in chlorophyll degradation (*SGR*, *NOL*, and *PAO*) and increased the expression levels of genes involved in chlorophyll biogenesis (*CHLH* and *CAO*). This indicates that the occurrence of severe cellular water deficit caused by drought-affected chlorophyll synthesis, Which accelerated the decomposition rate of the original chlorophyll and consequently decreased in chlorophyll content (Javadi et al., 2017; Li et al., 2024). In contrast, FA increases chlorophyll content by improving the water retention capacity of oat leaves, allowing the leaves to absorb more light energy, mitigating chlorophyll degradation, and promoting chlorophyll synthesis (Nikoogoftar-Sedghi et al., 2024; Li et al., 2024). This result further supports the expression of antenna proteins, particularly *LHCB1* (**Supplementary Figure 2**), whose genes are involved in the regulation of light capture in photosystem II (Bielczynski et al., 2020). FA may alleviate stress injury by regulating the light-capture protein of oat plants under drought stress. In wheat, the overexpression of *LHC* in chloroplast tissue enhances stress tolerance (Chen et al., 2023). The up-regulation of the photosynthesis-related protein *PsbR* further confirmed the role of FA in increasing photosynthetic efficiency of oat plants under drought stress (Wang et al., 2019) (**Supplementary Figure 2**). In conclusion, FA can enhance the drought tolerance and growth of oats by increasing chlorophyll, antenna protein, and photosynthetic pathway-related gene expression.

Elevated levels of H_2_O_2_ in plant tissues under drought stress may lead to oxidative stress, and the excessive accumulation of ROS leads to an increase in MDA, a key indicator of oxidative damage to cell membrane integrity (Begović et al., 2018). In this study, we found that H_2_O_2_ and MDA increased significantly under drought stress, but spraying FA inhibited this phenomenon. This indicates that drought stress led to the dehydration and large accumulation of H_2_O_2_ and MDA in the oat leaves, which ultimately led to the oxidative damage and growth retardation of leaves, while FA mitigated the oxidative damage of cell membranes by direct scavenging of H_2_O_2_ (**Figures 2A, B**) (Irani et al., 2021). The antioxidant enzyme defense system is another important mechanism for plants to manage drought stress, protecting the cell membrane from oxidative damage (Wang et al., 2023b). FA is a broad-spectrum plant growth regulator with pleiotropic effects on a wide range of unfavorable environmental factors (Sun et al., 2020; Liang et al., 2024). In our study, a significant increase in antioxidant enzyme activities was observed under water deficit conditions, and antioxidant enzyme activities were further increased by FA treatment (**Figures 2C-G**). This indicates that FA exerted free radical detoxification and cell membrane structure repair and protection effects on plant seedlings, This could maintain the stability of the intracellular environment, effectively alleviating the oxidative damage caused by drought stress, and enhancing the drought resistance of plant seedlings. Similar results were observed in cabbage seedlings under calcium nitrate stress (Wu et al., 2023). The phenylpropanoid biosynthesis pathway not only affects plant growth and development but also stress responses. Dong and Lin (2021) found that the plant phenylpropanoid metabolic pathway, especially lignin synthesis, has an important regulatory function in plant responses to biotic and abiotic stresses. Phenylpropanes are a large class of plant secondary metabolites. In the phenylpropanoid biosynthesis pathway, phenylalanine is deaminated by *PAL* to produce cinnamic acid, which is then converted to p-coumaric acid. Finally, p-coumaric acid is converted to p-coumaroyl coenzyme A catalyzed by 4-coumaroyl coenzyme A ligase (*4CL*), and p-coumarin and p-coumarol coenzyme A are ultimately formed into lignin through various metabolic pathways (Deng and Lu, 2017). In this study, we found that *PAL* genes were upregulated following the spraying of FA under drought stress, indicating that the biosynthesis of coumaric acid and ferulic acid should be higher (Chen et al., 2021). Interestingly, this study found that the levels of metabolites on coumaric acid and ferulic acid decreased under the action of FA, and more lignin synthase genes were also upregulated, such as *4CL*. Therefore, it is speculated that more p-coumaric acid and ferulic acid may be converted into lignin monomer sinapyl alcohol under the catalysis of *4CL*, which promotes the synthesis of lignin (Zhang et al., 2023). The increase in sinapyl alcohol in this study also confirms this phenomenon. Peroxidase (POD) is a multi-functional enzyme. It is involved in several different plant physiological processes, including stress resistance, oxidation, and the polymerization of lignin monomers after transportation to the cell wall (Lee et al., 2021). Under stress, plants enhance stress resistance by up-regulating the expression of genes encoding *POD* (Fei et al., 2020; Li et al., 2021). Research has shown that *POD* is a hub gene related to lignin biosynthesis (Yang et al., 2023). In this study, the upregulated expression of the *POD* gene further indicates that FA can promote lignin synthesis. Lignin is an important cell-wall component, and the increase in the lignin level contributes to the fixation of the cell wall (Shah et al., 2023). Plant lignification has been reported to improve stress resistance (Chun et al., 2019) and drought (Gu et al., 2020). Therefore, it is speculated that FA can help plants increase the thickness and strength of cell walls by promoting lignin synthesis, which controls water losses, reduces the wilting degree of plants, and enhances the drought resistance of oats (Ibrahim et al., 2019). The changes in PAL and POD activities in the physiological indexes further confirmed the possibility of lignin accumulation in plants.

Glutathione metabolism is known to be involved in the maintenance of cellular redox homeostasis under drought stress. This adaptive mechanism repairs oxidative damage by utilizing a variety of reactive oxygen scavengers (antioxidants) and redox reactions (Raihan et al., 2023; Wang et al., 2023c). Glutathione metabolism-related genes and metabolites regulate cellular redox homeostasis during abiotic stress (Yang et al., 2022; Horváth et al., 2023; Ali et al., 2024). For example, the overexpression of glutathione metabolism-related genes and proteins, including GST and G6PDH, improved drought tolerance in transgenic plants (Wang et al., 2016; Wei et al., 2020). The overexpression of *GST* activates its antioxidant-related transcripts, reduces ROS accumulation, and enhances plant drought resistance (Nerva et al., 2022; Niu et al., 2024). In this study, the genes encoding *GST* were all upregulated after spraying FA under drought stress, suggesting that FA may improve the scavenging capacity of ROS in oats under drought stress by increasing the expression of *GST*. This is similar to the results of a previous study on tea plants (Sun et al., 2020). The pentose phosphate pathway produces of NADPH, which promotes the production of reduced glutathione and thus maintains cellular redox homeostasis (Sharkey, 2021). G6PDH, as the rate-limiting enzyme in the pentose phosphate pathway (PPP), plays a key role in maintaining redox homeostasis (Li et al., 2023b). Under drought and cold stress, G6PDH maintains cellular redox homeostasis by increasing NADPH/NADP^+^ levels (Landi et al., 2016; Zhang et al., 2020). The expression of *G6PDH* can control the rate of increase of ROS and improve stress tolerance (Jiang et al., 2022). In this study, we found that the expression of the gene encoding *G6PDH* was downregulated after spraying with FA under drought stress. This suggests that glutathione reduction in FA-treated oat plants does not strictly depend on the positive PPP response to replenish NADPH for drought resistance. In contrast, Sun et al. (2020) showed that FA may enhance the ROS scavenging capacity of tea plants under moderate to severe drought stress by increasing the expression of *G6PDH* and the biosynthesis of the reduced form of glutathione, This differs from our results, possibly due to the differences in the species or the degree of stress employed in the experiments. Ascorbic acid is a powerful antioxidant that neutralizes free radicals and reduces oxidants, but in this process, ascorbic acid itself is oxidized to dehydroascorbic acid and loses its antioxidant function (Njus et al., 2020). Glutathione is a tripeptide that is able to reduce dehydroascorbic acid and turn it back into ascorbic acid, allowing it to continues to play an antioxidant role (Foyer and Kunert, 2024). In this study, the level of dehydroascorbic acid decreased after spraying FA under drought stress, and the results were consistent with the physiological level. This may be because the FA treatment accelerated the reduction process of glutathione and converted it back to ascorbic acid, which enhanced the antioxidant effect and alleviated the oxidative damage caused by drought stress to oats (Kim et al., 2020). As a ubiquitous amino acid, glutamate is involved in the synthesis of a wide range of precursors and plant resistance-related proteins or non-protein amino acids (Forde and Roberts, 2014). The exogenous application of glutamate is reported to enhances salt tolerance in lettuce (Franzoni et al., 2022). Glutamate treatment under drought stress significantly increases the proline content in the phloem and xylem of oilseed rape (La et al., 2020). Proline synthesis occurs in plants under two pathways, namely, through the glutamate and ornithine pathways. Among them, glutamate is directly or indirectly involved in altering certain physiological metabolisms (e.g., carbon and nitrogen metabolism) in plants by regulating the expression of related genes and the activities of key enzymes, which helps plants to grow under stress and enhances their adaptability to various adversities (Goto et al., 2020; Teixeira et al., 2020). In this study, FA treatment under drought stress increased the level of the metabolite glutamate. It is hypothesized that FA may promote proline synthesis and enhance drought resistance primarily by increasing the glutamate pathway.

## 5 Conclusion

**Figure 9:**
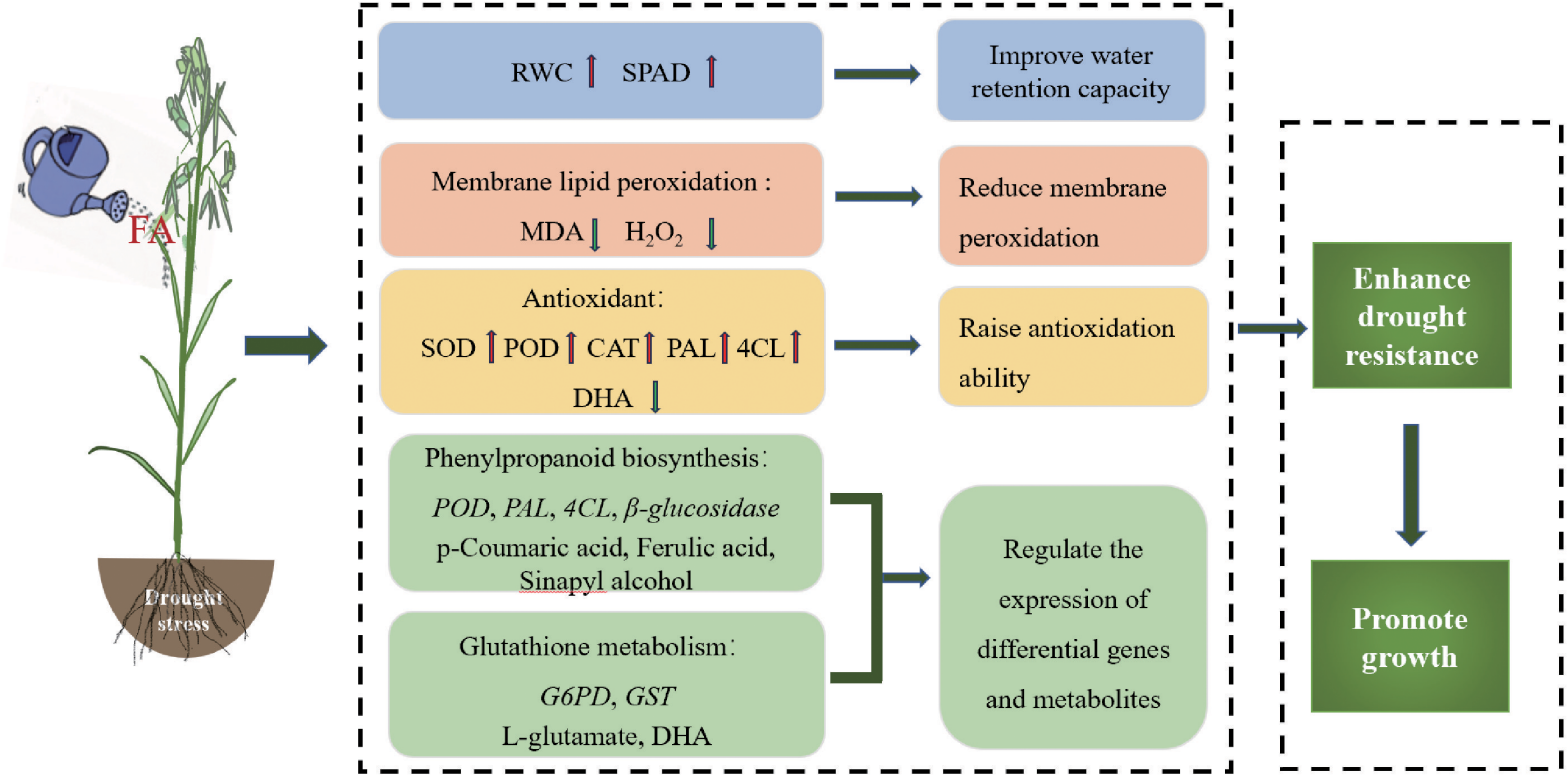
Mechanism of fulvic acid regulating drought resistance of oats.

This study indicates that FA improves drought tolerance and promotes the growth of oats through physiology and gene and metabolite expression under water-deficit conditions. FA reduces cell membrane damage and ROS accumulation, alleviates drought stress, and promotes oat growth by increasing chlorophyll content and enhancing antioxidant defense activity. It also improves the drought resistance of oats by inducing the expression of genes in phenylpropanoid biosynthesis, glutathione metabolism, and metabolite accumulation (**Figure 9**). Our findings revealed a new direction and theoretical basis for the future research on the use of FA technology to improve crop drought resistance.

## Data Availability

The datasets presented in this study can be found in online repositories. The names of the repository/repositories and accession number(s) can be found in the article/**Supplementary Material**.
