# Supplementary material for: Integrative transcriptome and metabolome analysis reveals the mechanism of fulvic acid alleviating drought stress in oat
Source: Front Plant Sci. 2024 Sep 19;15:1439747. doi: 10.3389/fpls.2024.1439747 (PMC11446754; doi:10.3389/fpls.2024.1439747)
Supplement: Supplementary file 1 [file DataSheet1.zip › Figures S1 - S3.DOCX]

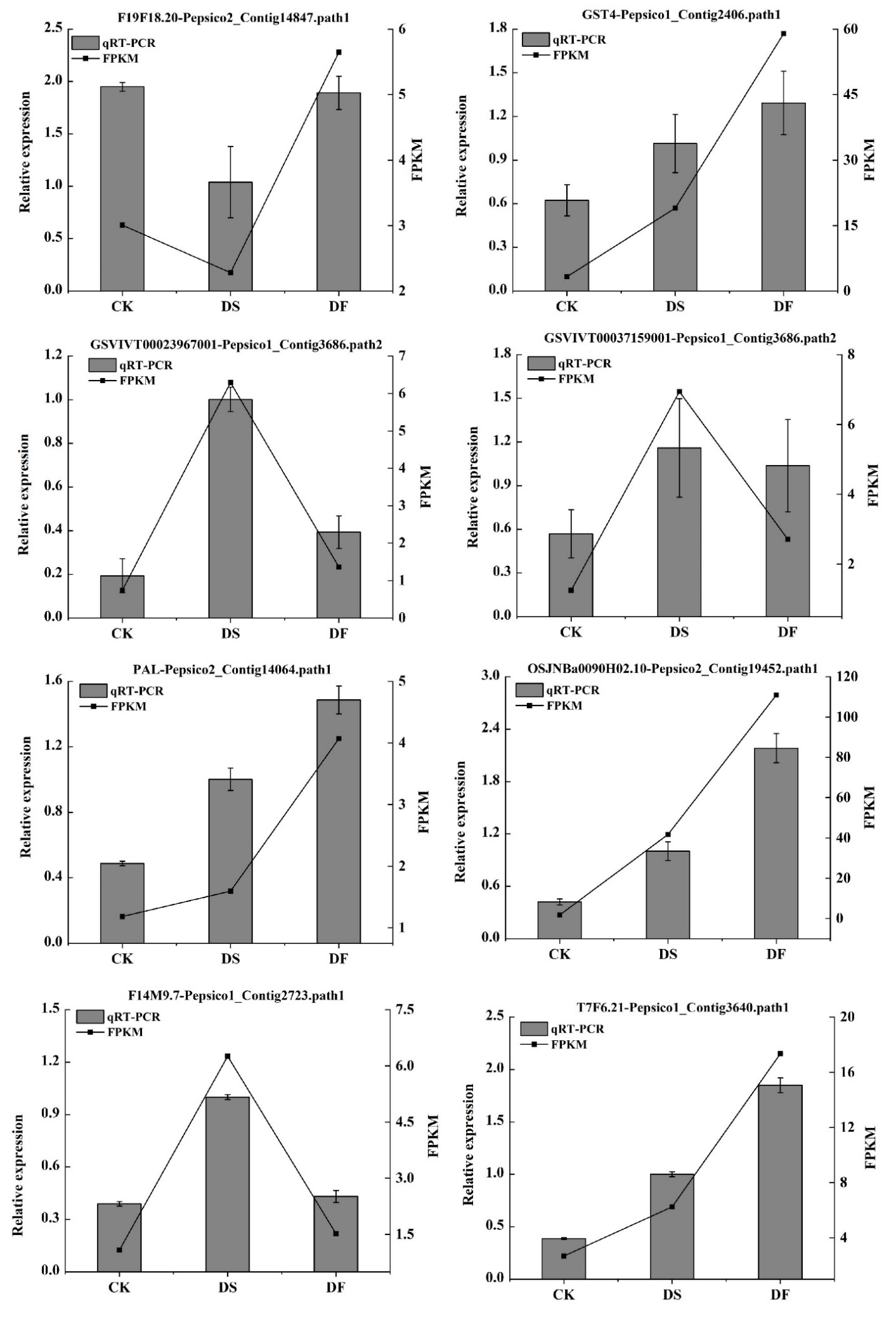


Figure S1. The qRT-PCR validation of 8 genes.


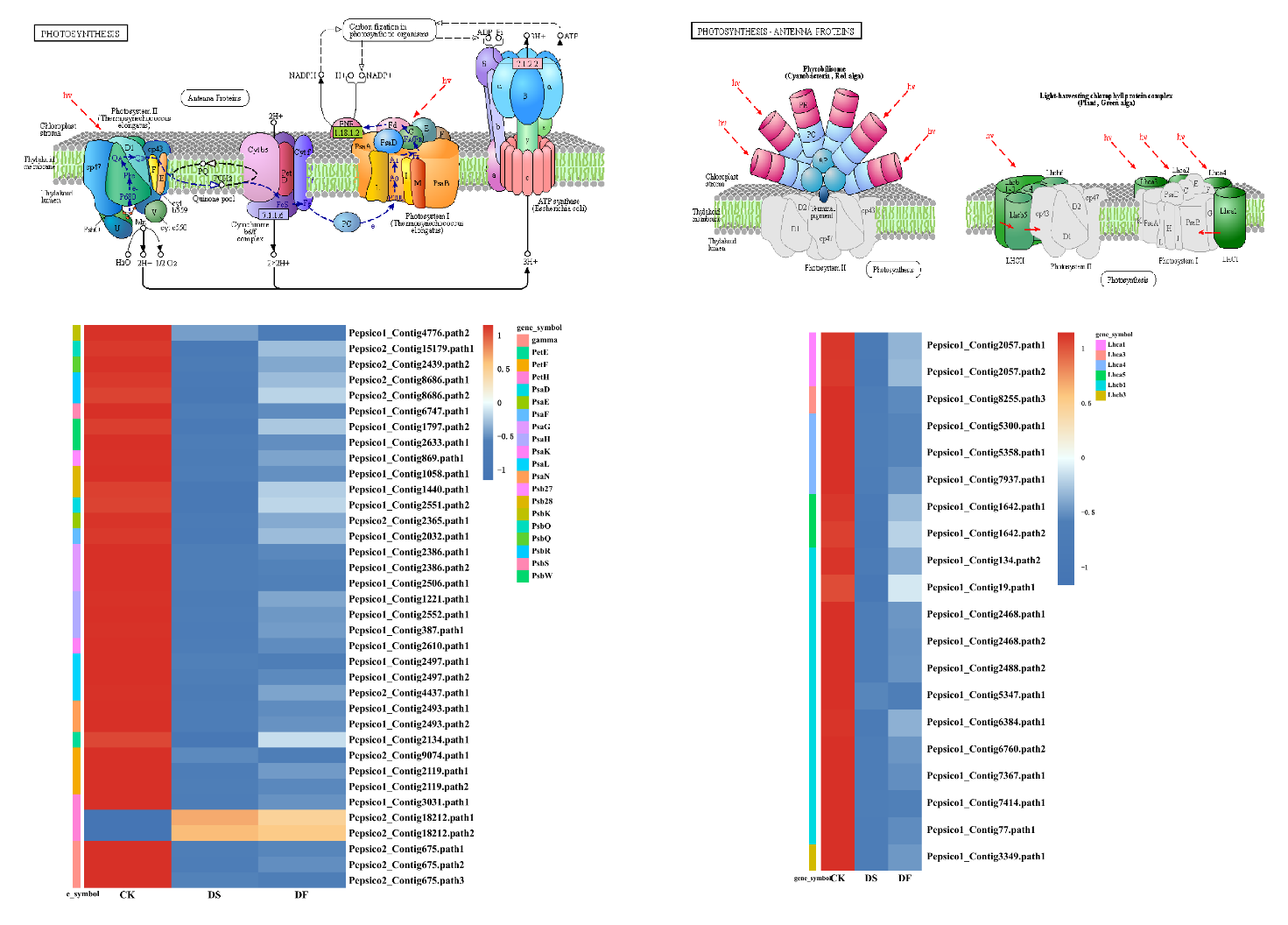


Figure S2. The DEGs involved in photosynthesis. The rectangular patterns represent the genes, and the heatmap at the corresponding place depicts the differential expression of each identified gene, which ranges from blue (low) to red (high).


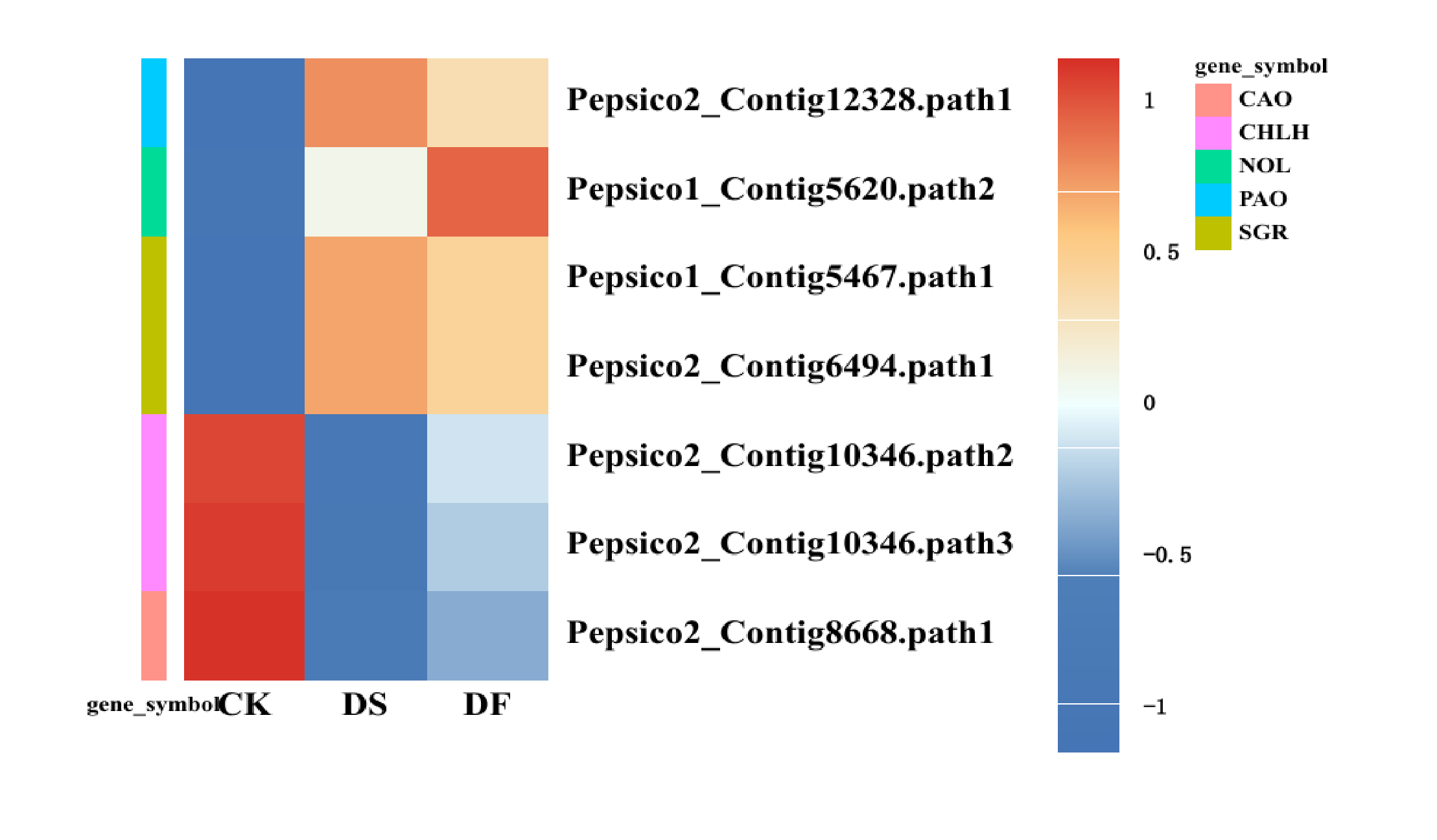


Figure S3. The DEGs involved in chlorophyll. The rectangular patterns represent the genes, and the heatmap at the corresponding place depicts the differential expression of each identified gene, which ranges from blue (low) to red (high).
